# MMP2 is a immunotherapy related biomarker and correlated with cancer-associated fibroblasts infiltrate in melanoma

**DOI:** 10.1186/s12935-023-02862-5

**Published:** 2023-02-14

**Authors:** Kunwei Peng, Yanyan Zhang, Deyi Liu, Jingqi Chen

**Affiliations:** 1 Guangdong Provincial Education Department Key Laboratory of Nano-Immunoregulation Tumour Microenvironment, Department of Medical Oncology, The Second Affiliated Hospital of Guangzhou Medical University, No. 250 Changgang East Road, Guangzhou, 510260 Guangdong People’s Republic of China; 2 Department of Infectious Diseases, Guangzhou First People’s Hospital, School of Medicine, South China University of Technology, Guangzhou, Guangdong People’s Republic of China; 3 Department of General Practice, The Second Affiliated Hospital of Guangzhou Medical University, Guangzhou, Guangdong People’s Republic of China; 4 Translational Medicine Center, The Second Affiliated Hospital of Guangzhou Medical University, No. 250 Changgang East Road, Guangzhou, 510260 Guangdong People’s Republic of China

**Keywords:** MMP2, Immunotherapy, Cancer associated fibroblasts, Melanoma

## Abstract

**Background:**

Mounting evidence supports that matrix metalloproteinase (MMPs) are highly associated with tumor progression and that targeting MMPs may overcome the barrier of immune suppression. Among these, whether MMP2 functions as an immunosuppressive role in melanoma, remains unclear.

**Methods:**

The Cancer Genome Atlas (TCGA) and Gene Expression Profiling Interactive Analysis 2 (GEPIA2) databases were used to assess the prognosis of MMP2 in melanoma, after which Tumor immune estimation resource (TIMER) was used to explore the relationship between MMP2 expression and cancer associated fibroblasts (CAFs) infiltration. Finally, we evaluated the efficacy of MMP2 inhibitor on CAFs infiltration and immunotherapy using a mouse melanoma model.

**Results:**

In general, the expression of MMP2, MMP13, MMP16, MMP17 and MMP25 were significantly associated with skin cutaneous melanoma (SKCM) patients prognosis, among which MMP2 low expression benefited patients the most. Especially, the overall survival (OS) of BRAF mutation patients with high MMP2 expression was significantly lower than the MMP2 low expression group, but there was no significant difference in BRAF wild-type patients. KEGG and GO enrichment analysis indicated that MMP2 related genes were mostly associated with extracellular structure organization, collagen-containing extracellular matrix and extracellular matrix structural constituent. Furthermore, in almost all cancers, MMP2 expression was positively correlated with CAFs infiltration. MMP2 inhibitor works synergistically with PD-1 antibody and induces tumor regression in a mouse melanoma model, which is dependent on decreased CAFs infiltration.

**Conclusions:**

This suggests that MMP2 plays a vital role in the regulation of CAFs infiltration, potentially participating in immunotherapy response, and thus representing a valuable target of immunotherapy in melanoma.

## Background

In the past few decades, skin cutaneous melanoma (SKCM) is the most aggressive skin cancer, which seriously endangered human health [1]. With a better understanding of the mechanism underlying SKCM occurrence and development, we have more options to treat it. For patients with advanced SKCM, the advent of targeted therapy and immune checkpoint inhibitors have dramatically evolutionized the treatment landscape [2–4]. Antibodies targeting the immune checkpoints, such as cytotoxic T-lymphocyte–associated antigen 4 (anti-CTLA-4) and programmed cell death protein-1 (anti-PD-1), some patients have achieved long-term benefits [5]. However, approximately 40 to 45% patients experience primary resistance to immune checkpoints blockade, and the 5-year survival rate of patients with advanced SKCM, remains low at only 39% [6–8]. The factors associated with primary resistance include inadequate T-cell infiltration, as well as immunosuppressive factors in the tumor microenvironment [9, 10]. Therefore, we need to screen for immune-related genes in SKCM to identify novel biomarkers and potential drug targets to improve melanoma immunotherapy.

Cancer-associated fibroblasts (CAFs) are one of the most abundant stromal components in the tumor microenvironment [11]. A large number of studies have shown that CAFs play an important role in cancer progression and have important clinical significance [12]. Mechanistically, CAFs establish and reshape the extracellular matrix (ECM) structure, enabling tumour cells to invade through the tumor microenvironment and interact with cancer cells or other stromal cells by secreting growth factors, cytokines and chemokines [13]. Additionally, CAFs promote establishment of an immunologically cold tumor phenotype, either by directly inhibiting the infiltration and activity of T cells or by promoting recruitment of other immunosuppressive cell types, and influence responses to treatment [14–16]. Undoubtedly, understanding the role of CAF will provide the basis for new strategies for immunotherapy.

Matrix metalloproteinases (MMPs) are a family of secreted peptidases critical for the degradation of extracellular matrix and basement membrane [17]. MMPs play pivotal roles in several aspects of cancer, such as cancer differentiation, invasion and metastasis through the proteolysis of structural extracellular matrix proteins [18]. Moreover, activated MMPs directly induce the epithelial mesenchymal transition in epithelial cells, lending to cancer progression [19]. Some MMPs overexpression (such as MMP2, MMP9 and MMP13) were directly associated with poorer prognosis in many cancers [20–23]. Mounting evidence support that MMPs were highly associated with the microenvironment during tumor progression and that targeting MMPs may overcome the barrier of immunosuppression [24, 25]. However, no study is available on whether MMP2 functions as an immunosuppressive biomarker in melanoma.

In this study, we evaluated the association between MMPs and melanoma prognosis using TCGA database, and found that MMP2 low expression benefited patients the most. Especially in BRAF mutation patients, the OS of patients with high MMP2 expression was significantly lower than patients with low MMP2 expression, but not in BRAF wild-type patients. Furthermore, MMP2 expression was positively correlated with CAFs infiltration in almost all cancers. Gene enrichment analysis indicated that MMP2 related genes were mostly associated with extracellular structure organization. According to a mouse melanoma model, we found that MMP2 inhibitor synergistically with PD-1 antibody induces tumor regression, which dependent on decreased infiltration of CAFs. Our results suggest that MMP2 maybe a promising therapeutic target for improving the efficacy of immunotherapy.

## Methods

### Survival and pathological features analysis

The Cancer Genome Atlas (TCGA) collected over 20,000 primary cancer and matched normal samples spanning 33 cancer types, include gene expression profiles, clinical data and somatic mutation data. We use UCSCXena (https://xenabrowser.net/) to download data of SKCM in the TCGA database. Firstly, we analyzed the relationship between the expression of MMP family genes and the prognosis of SKCM. Secondly, we utilized the “Survival Map” module of GEPIA2 (Gene expression profiling interactive analysis, version 2) to assess the relationship between MMP2 expression and patient outcomes across all TCGA tumors, including OS and DFS. The median of MMP2 expression was used as the cut-off value to distinguish low expression and high expression cohorts. The survival plots and log-rank p value were also obtained through the “Survival Analysis” module of GEPIA2. The univariate logistics regression was used to determine the association of MMP2 expression with SKCM pathological features.

### Tumor microenvironment and immunotherapy analysis

We explored the association between MMP2 expression and CAFs infiltration across all TCGA tumors by TIMER2 (Tumor immune estimation resource, version 2). The partial correlation value and p value were calculated by Spearman's rank correlation test after purity adjustment. Immune score and stromal score represented the abundance of immune components and stromal components in tumor microenvironment, respectively.

Immunotherapy data were obtained from GSE78220. The median of MMP2 expression was used as the cut-off value to distinguish low-expression and high-expression cohorts. Responses to immunotherapy include partial response (PR) and complete response (CR).

### Enrichment analysis

Gene Ontology (GO) enrichment analysis and Kyoto Encyclopedia of Genes and Genomes (KEGG) pathway enrichment analysis were performed to research the potential biological characteristics of differentially expressed genes by using R package of clusterProfiler. The GO analysis contains cellular component (CC), molecular function (MF), and biological process (BP). FDR < 0.05 was considered statistically significant.

### In vitro treatment studies

Female immunocompetent (C57BL/6) mice (aged 4–6 weeks) were purchased from the SPF (Beijing) Biotechnology, and maintained under specific-pathogen-free (SPF) grade. Mouse melanoma cells B16F10 were derived from C57BL/6 mice with strong proliferation ability and high tumorigenic rate. Wild-type B16F10 cells (5 × 10^5^) were injected subcutaneously into mice. Nearly 1 week later, the mice were pooled and randomly divided into the control or treatment groups. On day 12 following inoculation with B16F10 cells, mice were administered daily with MMP2 inhibitor (SB-3CT, 10 mg/kg, ip), anti-PD-1 antibody (100 μg/mouse/3 days, ip), combination therapy or control vehicle only, for 10 days. Tumor volume was calculated as tumor length × (tumor width)^2^ × 1/2. Subsequently, tumors were collected and analyzed by immunohistochemistry (IHC).

### Statistical analysis

Experimental data were expressed as mean ± SEM. Kruskal Wallis test analyzed statistical differences for variables of more than two groups, and two-sided Student’s t test was used in two groups. Two sides p < 0.05 was considered statistically significant, with the analysis and mapping by Graphpad Prism software 7. * indicates statistical significance: * indicates p < 0.05, ** indicates p < 0.01, *** indicates p < 0.001, ns indicates p > 0.05, that is, no statistical difference.

## Results

### The association between MMPs expression and SKCM patients prognosis

**Fig. 1:**
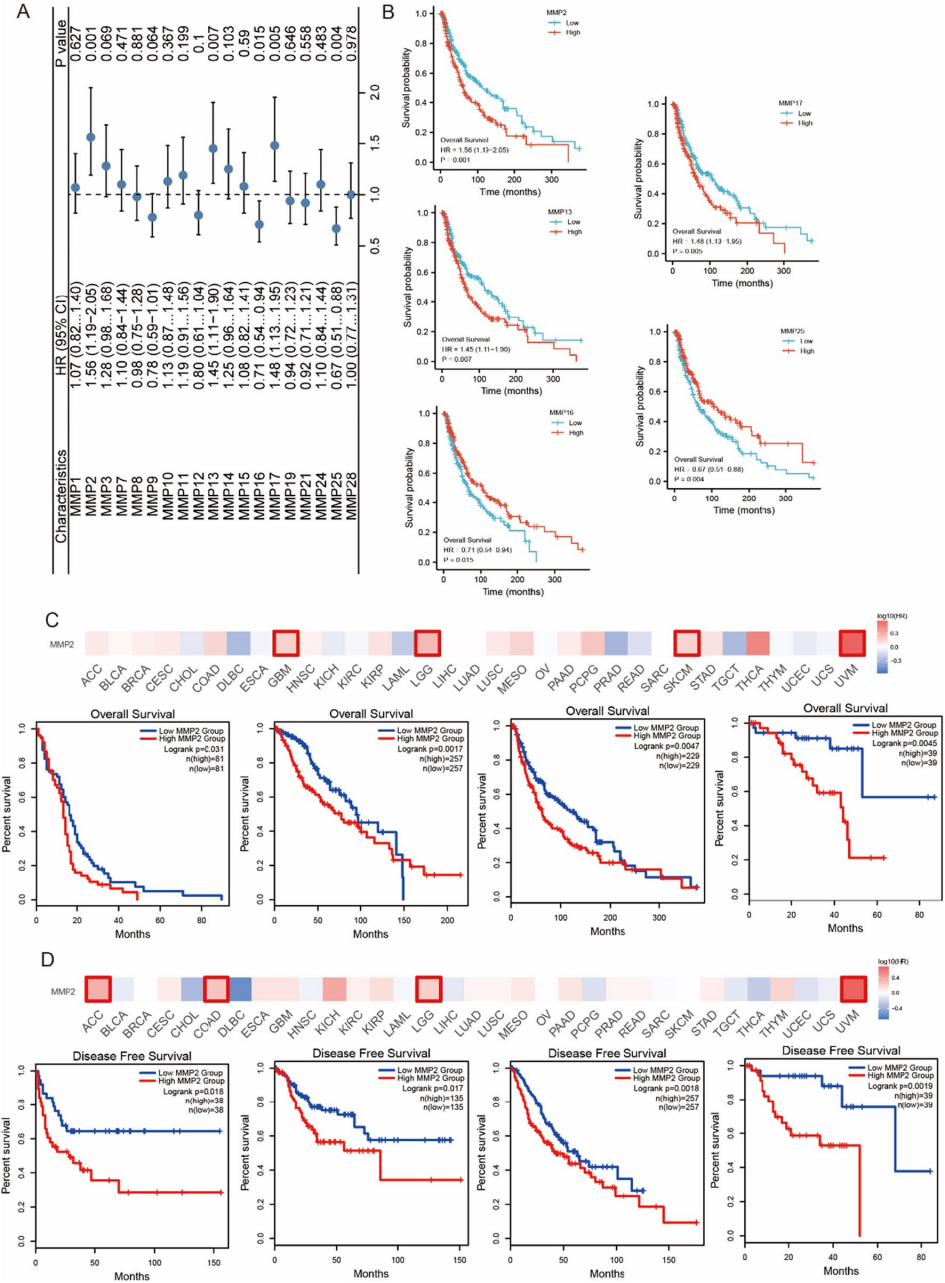
The impact of MMPs in SKCM survival. **A** Survival analysis of MMPs in SKCM. **B** Survival curves of MMP2, MMP13, MMP16, MMP17 and MMP25 in SKCM. **C** OS and **D** DFS analysis of MMP2 in different tumors by used GEPIA2. The survival map and Kaplan–Meier curves with positive results were given

To examine the prognostic significance of MMPs, TCGA RNA-seq and clinical data were used to evaluate the prognosis of MMPs in SKCM. We found that the expression of MMP2, MMP13, MMP16, MMP17 and MMP25 were significantly associated with SKCM patients prognosis, among which MMP2 low expression benefited patients the most (Fig. 1A, B). As shown in Fig. 1C, elevated MMP2 expression was significantly related to a poorer OS in GBM (p = 0.031), LGG (p = 0.0017), SKCM (p = 0.0047), and UVM (p = 0.0045). Increased MMP2 expression was associated with poorer DFS in ACC (p = 0.018), COAD (p = 0.017), LGG (p = 0.0018), and THYM (p = 0.019) (Fig. 1D). These results demonstrate that MMP2 expression significantly correlated with poor survival in SKCM.

### Relationship between MMP2 expression and clinicopathological parameters

**Fig. 2:**
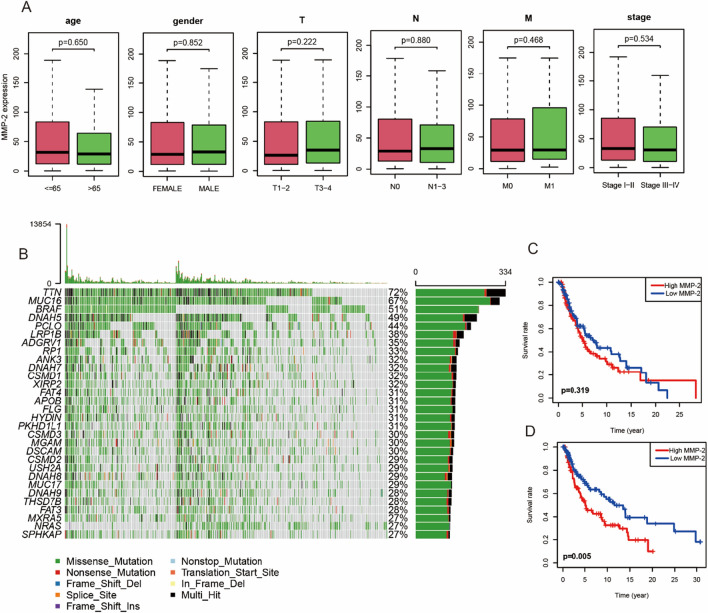
Relationship between MMP2 expression and clinical features. **A** The difference of MMP2 expression in patients with different clinical features. **B** Landscape of gene mutation in SKCM patients (top 30). **C**, **D** Survival curves of MMP2 in patients with BRAF mutation and BRAF wild type

In order to investigate the influence of various clinical features on MMP2 expression, we analyzed the differences of MMP2 expression in patients. In further studies, we found that age, gender, T stage, N stage, M stage and clinical stage had no significant influence on MMP2 expression (Fig. 2A). We then inspected the gene mutation frequency of SKCM in the TCGA database, and found that TTN, MUC16 and BRAF were the three most frequent mutation genes (Fig. 2B). In BRAF mutation patients, the OS of patients with MMP2 high expression group was significantly lower than MMP2 low group (Fig. 2C). In BRAF wildtype patients, MMP2 expression had no significant effect on survival (Fig. 2D), suggesting that MMP2 high expression was an adverse prognostic factor in BRAF mutant SKCM patients.

### MMP2 high expression correlates with CAFs infiltration in melanoma

**Fig. 3:**
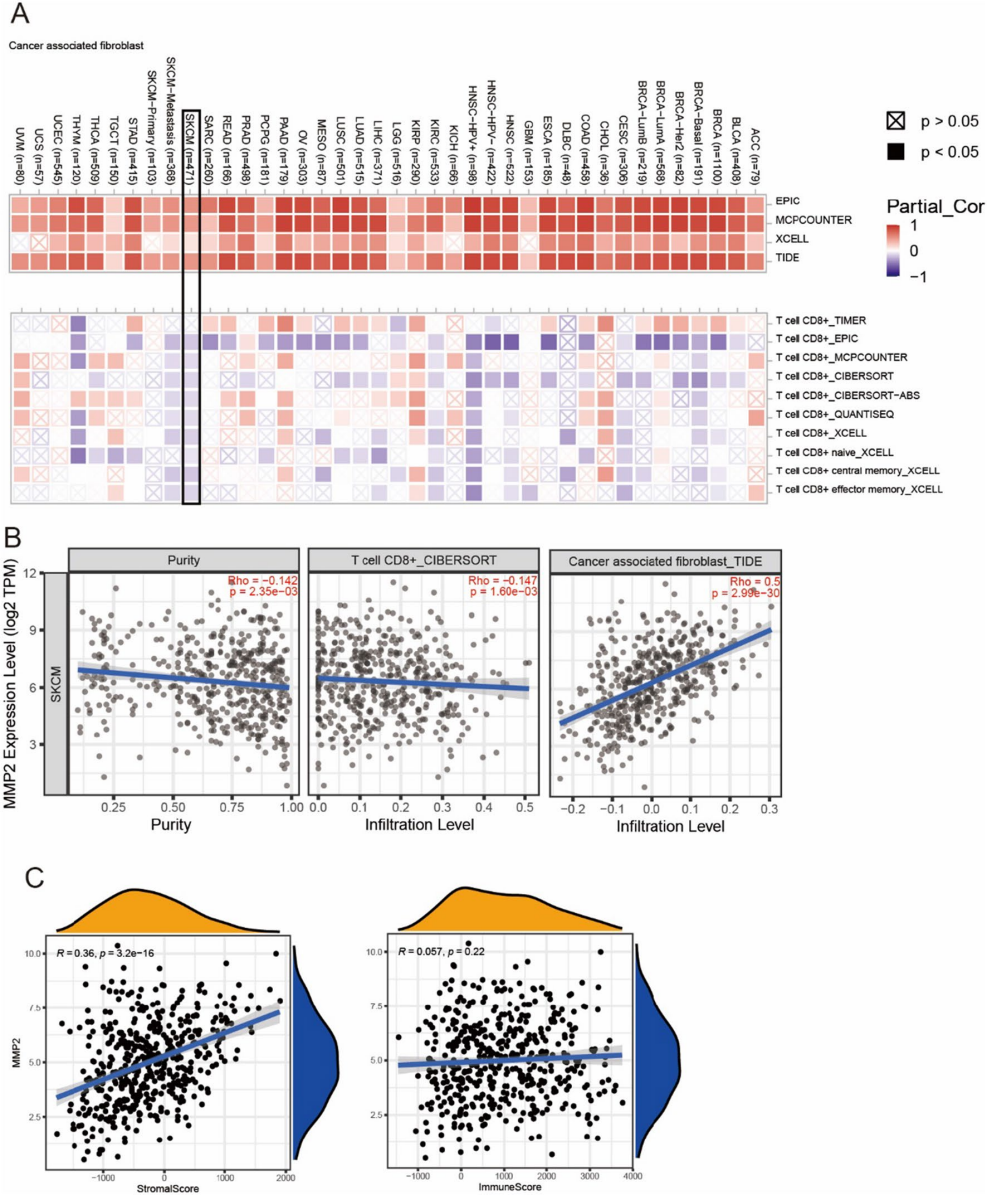
MMP2 expression correlates with CAFs infiltration. **A** Correlation between MMP2 expression and CAFs, CD8^+^ T cell infiltration across all types of cancer in TCGA. **B**, **C** Scatter plots of MMP2 expression and CAFs infiltration, CD8^+^ T cell infiltration, stromal score, immune score in SKCM

Tumor infiltrating immune cells and CAFs in the tumor microenvironment were reported to modulate immunotherapy [26, 27]. To explore whether MMP2 was involved in the process of CAFs infiltration, we first evaluated the association between MMP2 expression and CAFs infiltration across all types of cancer in TCGA. We observed a statistical positive correlation between MMP2 expression and CAFs infiltration based on all or most algorithms (Fig. 3A). However, the negative correlation was discovered between MMP2 expression and CD8^+^ T cell infiltration in SKCM (Fig. 3A). The scatterplot data of CAFs and CD8^+^ T cell infiltration in SKCM were presented in Fig. 3B. We further calculated the relationship between MMP2 expression and stromal score, immune score. Similarly, MMP2 was positively correlated with stromal score (Fig. 3C, R = 0.36, p = 3.2e−19) and negatively correlated with immune score (Fig. 3C, R = 0.057, p = 0.22). These results, to some extent, suggest that MMP2 was involved in the remodeling of SKCM tumor microenvironment.

### Enrichment analysis of MMP2-related partners

**Fig. 4:**
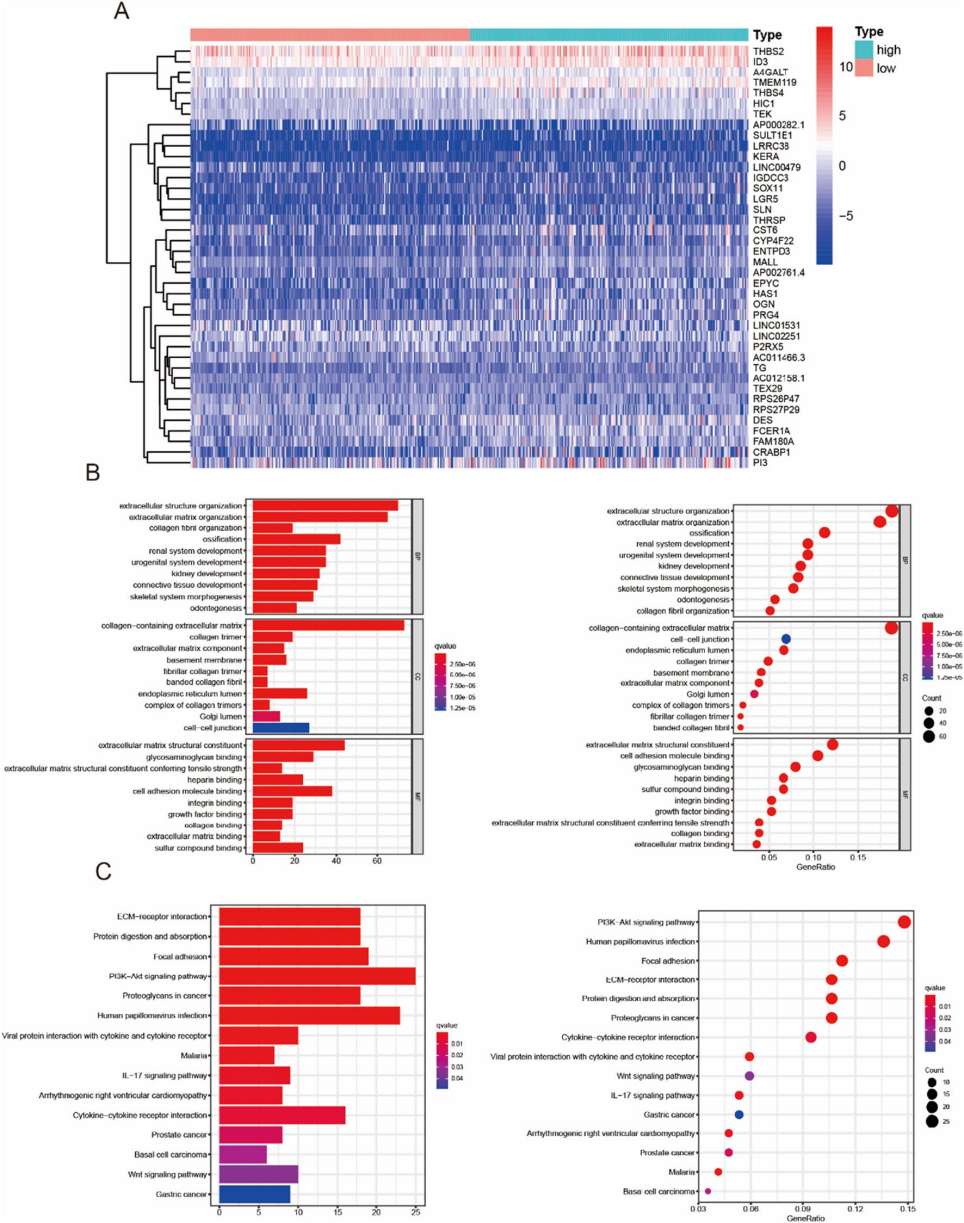
Functional annotation of MMP2 by GO and KEGG. **A** Top 20 differentially expressed genes in heatmap plot between MMP2-low and MMP2-high group. **B**, **C** GO and KEGG pathway analysis of differentially expressed genes associated with MMP2 expression

Further studies on the differentially expressed genes associated with MMP2 in SKCM, we ranked the significant differentially expressed genes between the MMP2-low group and MMP2-high group. Heatmap analysis showed the top 20 up-regulated and down-regulated genes (Fig. 4A). We used differentially expressed genes to perform KEGG and GO enrichment analyses. Specifically, MMP2 related genes were mostly associated with extracellular structure organization, collagen-containing extracellular matrix and extracellular matrix structural constituent (Fig. 4B). Meanwhile, the GO enrichment analysis indicated similar results, these genes were closely linked to the pathways or cellular biology of extracellular matrix metabolism, such as ECM (extracellular matrix) receptor interaction and protein digestion and absorption (Fig. 4C). These results indicated that MMP2 plays an important role in tumor microenvironment remodeling.

### Inhibition of MMP2 improves tumor microenvironment and enhances immunotherapy sensitivity

**Fig. 5:**
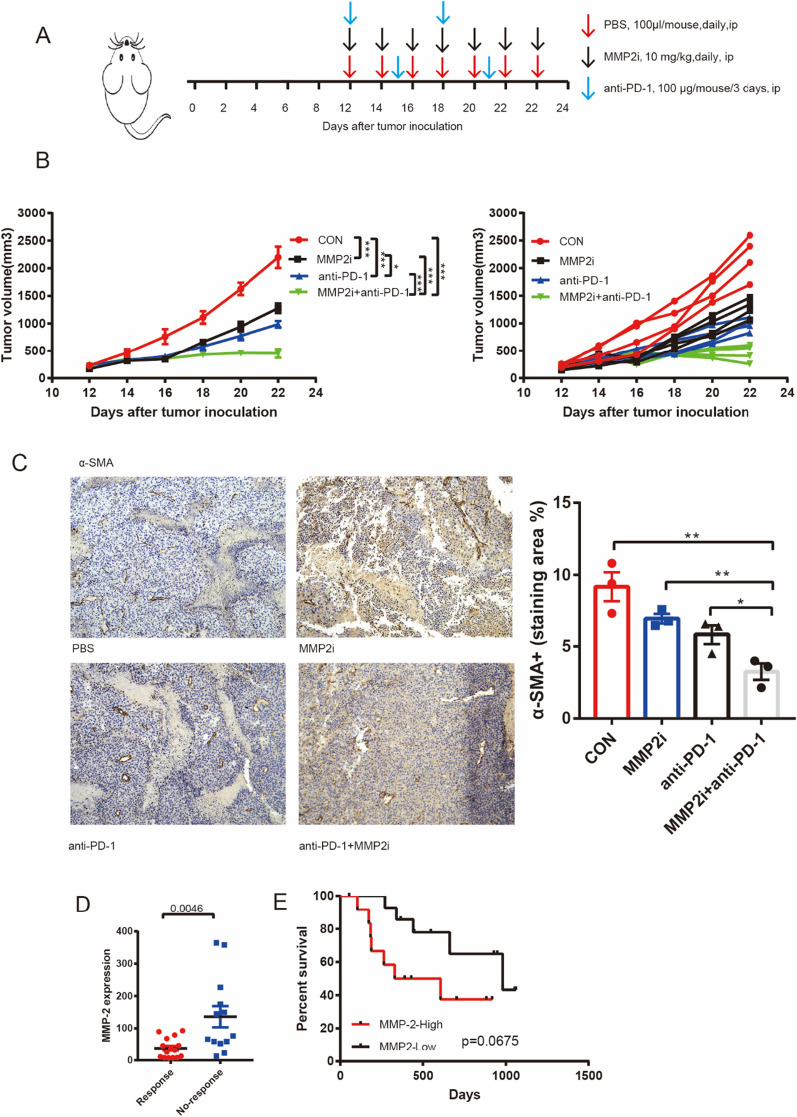
Synergistic therapeutic effect of MMP2 inhibitor and anti-PD-1. **A** Therapeutic plan of B16F10 tumor-bearing mice. **B** Tumor volumes of B16F10 tumor-bearing mice treated with control vehicle (PBS, 100 μl/mouse, daily, ip), MMP2 inhibitor (10 mg/kg, daily, ip), anti-PD-1 (100 μg/mouse/3 days, ip), or combination therapy, data are expressed as the mean ± SEM, n = 4 per group. **C** Representative α-SMA IHC staining of the tumor tissue and quantitation of α-SMA-positive fraction, data are expressed as the mean ± SEM, n = 3 per group. **D** MMP2 expression level in immunotherapy response group and no-response group. (E) Survival analysis of melanoma patients treated with immunotherapy

With the evidence that MMP2 expression increased CAFs infiltration, we further investigated whether MMP2 inhibitor has synergistic efficacy with anti-PD-1 therapy in a mouse model of melanoma. In brief, mice were subcutaneously injected with B16F10 cells. Then, mice were randomly divided into four groups when the tumor size reached about 200 mm^3^. We treated mice with MMP2 inhibitor, anti-PD-1 antibody, combination therapy or control vehicle only. Anti-PD-1 therapy was administered every 3 days and MMP2 inhibitor was administered daily, and tumor volume was measured every 2 days (Fig. 5A). In this experimental model, treatment with either anti-PD-1 antibody or MMP2 inhibitor alone significantly reduced tumor growth, while a combination treatment of MMP2 inhibitor and anti-PD-1 antibody achieved the best efficacy (Fig. 5B). To further verify that MMP2 inhibitor limited CAFs infiltration, we collected tumor tissues to study the tumor microenvironment of B16F10 tumor-bearing mice treated with MMP2 inhibitor and anti-PD-1 antibody combined or alone. Immunohistochemical (IHC) staining showed that the combination treatment decreased CAFs infiltration in the tumor microenvironment (Fig. 5C). Taken together, these results demonstrated that MMP2 inhibitor and anti-PD-1 antibody has synergistic efficacy and reduced CAFs infiltration.

Based on several published clinical data summarized in TISIDB, there was a significant difference in MMP2 expression between melanoma immunotherapy response and no-response. The expression level of MMP2 was significantly lower in patients who respond to immunotherapy than who did not respond (Fig. 5D). Patients with low MMP2 expression had longer survival, although the difference was not statistically significant (p = 0.0675, Fig. 5E). This suggested a potential prognostic value of MMP2 expression with melanoma immunotherapy.

## Discussion

Malignant transformation of melanocytes into metastatic melanoma is a multifactorial process, among which tumor microenvironment related factors play an important role [28]. Previous studies have initially investigated the differential expression of MMPs in benign and malignant tumors and the genes that may have prognostic value. In the present study, we assessed the prognostic value of MMPs in SKCM, and found that MMP2 low expression benefited patients the most. Especially in BRAF mutation patients, the OS of patients with high MMP2 expression was significantly lower than patients with low MMP2 expression, but there was no significant difference in BRAF wild type patients. Gene enrichment analysis indicated that MMP2 related genes were mostly associated with extracellular matrix structural constituent. Furthermore, in almost all cancers, MMP2 expression was positively correlated with CAFs infiltration. In the GEO database, high MMP2 expression predicts a lower immunotherapy response rate in melanoma and may serve as a predictor for immunotherapy. According to a mouse melanoma model, we found that MMP2 inhibitor synergistically with PD-1 antibody induces tumor regression, which dependent on decreased CAFs infiltration. Our results suggest that MMP2 maybe a promising therapeutic target of immunotherapy.

MMPs regulate ECM by degrading various components of ECM, thus affecting physiological functions such as cell adhesion, angiogenesis, apoptosis, proliferation, metastasis and epithelial-mesenchymal transformation [29]. In this regard, MMPs are able to proteolytically process substrates in the extracellular milieu and, in so doing, promote tumor progression. By contrast, elevated levels of MMPs are involved in pathologic processes such as arthritis [30], cardiovascular disease [31], neurodegenerative diseases [32] and cancer [33]. The elevated expression of MMP2 on tumor cells or stromal cells in the tumor microenvironmen was involved in tumor invasion and progression, with patients often having poorer prognosis [34, 35]. In our study, we evaluated the prognostic value of MMP2 expression in melanoma, the OS of patients with high MMP2 expression was significantly lower than patients with low MMP2 expression, and this trend was more pronounced in patients with BRAF mutation. Furthermore, in a melanoma immunotherapy cohort study, patients who respond to immunotherapy had significantly lower MMP2 expression than patients who did not respond. The survival time of patients with low MMP2 expression was longer, but the difference was not statistically significant, possibly due to the small number of cases. Together, these results suggest that MMP2 may be valuable as a prognostic biomarker in SKCM.

Biologically speaking, MMP2 may play an important role in immune response. Many studies have reported the interaction between MMP2 and immune cells. On the one hand, MMP2 may directly affect immune cells, for example, MMP2 conditioned dendritic cells promoted the differentiation of naïve CD4^+^ T cell into an inflammatory Th2 phenotype, via the MMP2 dependent cleavage of type-I IFN receptor and the subsequent decrease in IL-12 production [36]. Natural killer cells mediated cytotoxicity against cancer cell was reduced when exposed to MMP2 [37]. Macrophages may increase MMP2 production after IL-10 stimulation [38]. On the other hand, melanoma cells cross-present secreted MMP2 to human leukocyte antigen A*0201-restricted T cells in an αvβ3-dependent manner [39]. Therefore, MMP2 inhibits the function of immune cells and acts as an autoantigen to evade immune surveillance. Meanwhile, we found that the level of MMP2 expression correlated with CAFs infiltration and negatively correlated with CD8^+^ T cell infiltration. CAFs was one of the most abundant stromal cell types in the tumor microenvironment. Classically, CAFs were assigned with stimulating tumor growth and progression, and thus thought to play an essential role in tumor immunosuppression [40–42]. Similarly, our results also corroborated that MMP2 plays an immunosuppressive role in antitumor immunity. In a mouse model of melanoma, MMP2 inhibitor synergistically with PD-1 antibody induces tumor regression. IHC staining showed that the combination treatment decreased CAFs infiltration in the tumor microenvironment. Taken together, these results demonstrated that MMP2 inhibitor and anti-PD-1 antibody had synergistic efficacy and reduced CAFs infiltration.

Because of the pro-oncogenic functions, MMP2 has long been considered an attractive therapeutic target. In clinical trials, MMP inhibitors have failed to improve OS or alleviate symptom progression of cancer, mainly due to the non-specific of the drug and the complex background of MMP-specific effects [43]. In order to optimize the role of MMP inhibitors in cancer treatment, it is necessary to understand the role of MMP in carcinogenesis. However, MMP inhibitors may have a promising application in immunotherapy. In a study of breast cancer, treatment with the MMP inhibitor enhances the efficacy of the anti-CTLA-4 antibody on cancer metastasis [24]. In another study, MMP2 inhibitor modulates tumor immune surveillance and enhances the efficacy of immunotherapy by regulating PD-L1 [44]. In our study, treatment with MMP2 inhibitor alone significantly reduced tumor growth, while the combination of MMP2 inhibitor and anti-PD-1 antibody has a synergistic efficacy. More importantly, SB-3CT acts as an MMP2 inhibitor, targeting MMP2 rather than directly targeting the immune response, which may result in fewer toxic or immune-related adverse events, making SB-3CT a potential agent for future immunotherapy [45].

In conclusion, we applied comprehensive bioinformatics analysis to suggest that MMP2 mediate CAFs infiltration and impact patient prognosis in SKCM along with animal experiment verification. Our results highlighted that MMP2 expression was highly involved in immunotherapy response. Targeting MMP2 in combination with immunotherapy may be a novel therapeutic approach suppressing tumors, especially in SKCM. Further investigation is required to validate these findings.

## Data Availability

The public data used in this study can be found in online databases and the names of databases were provided in the paper. All other information can be obtained from the corresponding author upon reasonable request.
